# Structure of the *Triatoma virus* capsid

**DOI:** 10.1107/S0907444913004617

**Published:** 2013-05-14

**Authors:** Gaëlle Squires, Joan Pous, Jon Agirre, Gabriela S. Rozas-Dennis, Marcelo D. Costabel, Gerardo A. Marti, Jorge Navaza, Stéphane Bressanelli, Diego M. A. Guérin, Felix A. Rey

**Affiliations:** a Laboratoire de Virologie Moléculaire et Structurale, CNRS, 1 Avenue de la Terrasse, 91198 Gif-sur-Yvette CEDEX, France; b Fundación Biofísica Bizkaia, Barrio Sarriena S/N, 48940 Leioa, Bizkaia (FBB), Spain; c Unidad de Biofísica (UBF, CSIC, UPV/EHU), PO Box 644, 48080 Bilbao, Spain; d Departamento de Biología, Bioquímica y Farmacia, U.N.S., San Juan 670, (8000) Bahía Blanca, Argentina; e Departamento de Física, Grupo de Biofísica, U.N.S., Avenida Alem 1253, (8000) Bahía Blanca, Argentina; f Centro de Estudios Parasitológicos y de Vectores (CEPAVE-CCT, La Plata, CONICET-UNLP), Calle 2 No. 584, (1900) La Plata, Argentina

**Keywords:** *Triatoma virus*, TrV, *Cripavirus*, *Dicistroviridae*, virus structure, X-ray crystallography

## Abstract

The members of the *Dicistroviridae* family are non-enveloped positive-sense single-stranded RNA (+ssRNA) viruses pathogenic to beneficial arthropods as well as insect pests of medical importance. *Triatoma virus* (TrV), a member of this family, infects several species of triatomine insects (popularly named kissing bugs), which are vectors for human trypanosomiasis, more commonly known as Chagas disease. The potential use of dicistroviruses as biological control agents has drawn considerable attention in the past decade, and several viruses of this family have been identified, with their targets covering honey bees, aphids and field crickets, among others. Here, the crystal structure of the TrV capsid at 2.5 Å resolution is reported, showing that as expected it is very similar to that of *Cricket paralysis virus* (CrPV). Nevertheless, a number of distinguishing structural features support the introduction of a new genus (*Triatovirus*; type species TrV) under the *Dicistroviridae* family. The most striking differences are the absence of icosahedrally ordered VP4 within the infectious particle and the presence of prominent projections that surround the fivefold axis. Furthermore, the structure identifies a second putative autoproteolytic DDF motif in protein VP3, in addition to the conserved one in VP1 which is believed to be responsible for VP0 cleavage during capsid maturation. The potential meaning of these new findings is discussed.

## Abstract 2

The crystallographic structure of TrV shows specific morphological and functional features that clearly distinguish it from the type species of the *Cripavirus* genus, CrPV.

## 1. Introduction   

*Triatoma virus* (TrV) is a pathogen of *Triatoma infestans* and other related bloodsucking insects (also called ‘kissing bugs’) that are vectors of Chagas disease. This disease is a major health problem in Latin America, where it is endemic and affects about eight million people, 30–40% of whom develop cardiomyopathy and/or digestive megasyndromes (Rassi *et al.*, 2010 ▶). The aetiological agent of Chagas disease is the protozoan parasite *Trypanosoma cruzi*, which infects the insect vector, which in turn infects vertebrate hosts during blood meals. Increasing human population movements have resulted in Chagas disease also becoming an emerging health problem outside the countries in which it is endemic. Efficient vector-control programs based on spraying chemical insecticides are important to reduce insect populations in endemic areas. In an effort to identify entomopathogens as biological weapons against the vector, TrV was isolated from *T. infestans* (Muscio *et al.*, 1987 ▶), the main species transmitting Chagas disease in the southern countries of Latin America (Zeledón & Rabino­vich, 1981 ▶).

Biological control of insect pests is gaining importance in efforts to reduce the use of chemical pesticides and to reduce their residues in the environment, and also to overcome the development of pesticide resistance (Sonoda *et al.*, 2009 ▶; Lardeux *et al.*, 2010 ▶). Two different approaches are used for the biological regulation of pest populations: the large-scale release of genetically modified individuals and the release into the environment of natural enemies such as bacteria, fungi or viruses. Insect crop plagues can be dealt with by intervening directly in plantation areas, where the targets can easily be reached. In contrast, insect vectors that transmit human diseases are more difficult to reach because they inhabit wide regions covering human dwellings, both in urban and in rural or sylvatic areas. In addition to Chagas disease, tropical diseases such as malaria, dengue fever, African sleeping sickness and leishmaniasis constitute major challenges for the development of efficient biological control strategies. In the case of dengue virus (DV), which is transmitted by the mosquitoes *Aedes aegypti* and *A. albopictus* (Harris *et al.*, 2011 ▶), several potential control methods have been designed to either reduce the overall mosquito-vector population or to replace it with genetically modified populations that are unable to transmit the virus. One population-reduction strategy involved the use of natural or genetically engineered densoviruses that are pathogenic to *A. aegypti* mosquitoes (Carlson *et al.*, 2000 ▶). Similarly, different strains of the maternally inherited bacterium *Wolbachia* (a member of the Rickettsiales order) have been used to control *A. albopictus* mosquitoes (Blagrove *et al.*, 2012 ▶; Pan *et al.*, 2012 ▶) as well as the malaria vector *Anopheles gambiae* (Jin *et al.*, 2009 ▶). Viruses that have been used to control pests in agriculture and forestry include members of the *Baculoviridae* family, which are large DNA viruses (Szewczyk *et al.*, 2006 ▶), and also small insect RNA viruses (Harrap *et al.*, 1966 ▶; Moore, 1991 ▶).

TrV belongs to the *Dicistroviridae* family of small RNA viruses. A number of viruses of this family are pathogens of agricultural insect plagues such as grasshoppers (*Homalodisca coagulata*), olive fruit flies (*Dacus oleae*) and the red fire ant (*Solenopsis invicta*). Like TrV, these viruses have been considered as potential biopesticides (Hunnicutt *et al.*, 2006 ▶; Czibener *et al.*, 2000 ▶; Manousis & Moore, 1987 ▶; Bonning & Miller, 2010 ▶; Gordon & Waterhouse, 2006 ▶). The viruses in the *Dicistroviridae* family were previously known as ‘picorna-like viruses’ because of their similarity to the members of the *Picornaviridae* family of viruses that infect humans and other mammals. Both families are characterized by an icosahedral capsid of pseudo-triangulation number *P* = 3 and the absence of a lipidic envelope. The genome is a single-stranded positive-sense RNA (psRNA) molecule, which in the case of the *Dicistroviridae* is a bicistronic messenger RNA. The 5′ open reading frame (ORF) codes for a polyprotein precursor of the nonstructural proteins, including the viral polymerase, protease and helicase. The 3′ ORF codes for polyprotein P1, which is the precursor of the capsid proteins VP1, VP2, VP3 and VP4. The P1 polyprotein precursor contains these proteins in the order N-terminus–VP2–VP0–VP1–C-terminus, with a strictly conserved glutamine residue marking the C-­termini of VP2 and VP0, where it signals P1 cleavage, presumably by the viral 3C^pro^ protease as in picornaviruses. VP0 includes the mature proteins in the order N-terminus–VP4–VP3–C-terminus and appears to undergo cleavage into its component proteins *via* a capsid autoproteolytic mechanism upon RNA encapsidation (Agirre *et al.*, 2011 ▶). This process involves a conserved DDF motif present in VP1, as suggested for *Cricket paralysis virus* (CrPV; Tate *et al.*, 1999 ▶), the type species of the *Cripavirus* genus within the *Dicistroviridae* family. The crystal structure of the CrPV capsid allowed a detailed comparison with the structures of the picornaviruses, revealing specific differences, such as strand-swapping of the corresponding N-terminal tails of VP2 about the icosahedral twofold axes, which are specific to dicistroviruses (Tate *et al.*, 1999 ▶). In an effort to similarly characterize the TrV particle, we have crystallized and determined the three-dimensional structure of the virion at 2.5 Å resolution. We provide a detailed analysis of the structure in comparison to that of the CrPV capsid, showing that TrV differs substantially in surface features, in the absence of an ordered VP4 molecule in the capsid interior and in the presence in VP3 of a structurally conserved catalytic motif involving the same DDF sequence as in VP1. We also discuss putative regions of interaction with the host during entry, opening the way to functional studies to better understand the biology of TrV.

## 2. Materials and methods   

### 2.1. TrV purification, crystallization and structure solution and refinement   

TrV was purified from infected *T. infestans* bugs as described in a previous report (Rozas-Dennis *et al.*, 2004 ▶). Crystals were obtained under many different conditions and after optimization the best crystals were either rhombohedral (type I) or orthorhombic (type II) (Rozas-Dennis *et al.*, 2004 ▶). A stabilization process was carried out in order to improve vitrification for cryocooling: 5 µl of a stabilizing solution composed of 20%(*w*/*v*) PEG 2000 MME, 500 m*M* KCl, 100 m*M* Tris pH 8.5, 30%(*v*/*v*) glycerol was pipetted over the drop containing the crystals. This process was repeated three times, leaving a 2 min lapse between transfers, so that the crystals were subjected to increasing concentrations of cryoprotectant. The cryocooled crystals diffracted well and were highly resistant to radiation damage. Data from the two crystal forms were collected on beamline ID14-1 at the ESRF, Grenoble, France. An initial model for the TrV structure was obtained by molecular replacement with the *AMoRe* program (Navaza, 2001 ▶) using data to 4 Å resolution collected from the rhombohedral crystal form. The search model was a full capsid of 60 protomers of a polyalanine version of CrPV VP1, VP2, VP3 and VP4 (PDB entry 1b35; Tate *et al.*, 1999 ▶). The three best solutions had an *R* factor of 45% and a correlation coefficient of 30% and were related by the crystallographic threefold symmetry axis, indicating that the virus lies on this axis with one third of the capsid in the asymmetric unit. This confirms the analysis of the self-rotation function as described in Rozas-Dennis *et al.* (2004 ▶). The next best solution had an *R* factor of 49% and a correlation coefficient of 21%. Phasing was performed with the *CCP*4 suite of programs (Winn *et al.*, 2011 ▶). One third of the polyalanine CrPV capsid corresponding to the best solution was used for initial phasing to 7 Å resolution. Phases were improved and extended to 3.2 Å resolution by 20-fold density averaging and solvent flattening with *DM* in 100 steps (Cowtan, 1994 ▶). The resulting map was readily interpretable, with many side chains clearly visible, and a protomer of TrV VP1, VP2 and VP3 was built. The model of 20 protomers was refined to 3.2 Å resolution with *CNS* using NCS constraints (Brünger *et al.*, 1998 ▶). The test set was selected in thin shells to avoid correlation between *R*_free_ and *R*_cryst_ as much as possible. The matrices relating the 19 implicit protomers to the explicit protomer were refined at this stage and a final round of refinement of the rhombohedral form was performed. One half of the resulting TrV capsid was used as a search model for molecular replacement with the data collected from the orthorhombic form (see Table 1 ▶), as the self-rotation function indicated that in this form the virus lies on the crystallographic twofold symmetry axis. There was a single unambiguous solution that was further refined with NCS constraints to produce the final structure of *Triatoma virus* capsid at 2.5 Å resolution (see Table 1 ▶). 1% of reflections were selected in thin shells for the test set. The final crystallographic and free *R* factors of 17.12 and 17.32%, respectively, indicate that the final *R*_free_ is biased (Fabiola *et al.*, 2006 ▶).

### 2.2. Homology modelling   

A theoretical atomic model of VP4 from TrV was generated using the *MODELLER* software (Sali & Blundell, 1993 ▶; Eswar *et al.*, 2006 ▶). In order to provide all of the neighbouring contacts as a context for modelling, we employed template atomic coordinates of five copies of VP4 from a CrPV penton (previously superimposed on its TrV counterpart) combined with the coordinates of a VP1–VP3 TrV penton. Symmetry mates for the atomic model of the CrPV protomer (PDB entry 1b35; Tate *et al.*, 1999 ▶) and its TrV equivalent (PDB entry 3nap) were generated with *O* (Jones *et al.*, 1991 ▶) using the first five VIPER matrices for each structure (Carrillo-Tripp *et al.*, 2009 ▶), which contain the required transformations to build a penton; after joining all of the resulting structures, they were superimposed using *LSQMAN* and their chain IDs were normalized using *MOLEMAN*2 (Kleywegt & Jones, 1994 ▶). The alignment supplied to *MODELLER* was calculated using *T-Coffee* running with default parameters (Notredame *et al.*, 2000 ▶).

### 2.3. Sequence alignment   

A comparative study was performed by aligning the amino-acid sequences of the three major coat proteins from the following dicistroviruses: TrV, HiPV, PSIV, BQCV, CrPV and DCV. The criterion employed for the selection of the viruses was based on the availability of experimental data delimiting the N-termini of the coat proteins, although the members of the proposed *Aparavirus* genus were excluded for being excessively divergent in terms of sequence similarity. The alignment was performed using the *Expresso* mode of the *T-­Coffee* package (Di Tommaso *et al.*, 2011 ▶; Armougom *et al.*, 2006 ▶; O’Sullivan *et al.*, 2004 ▶; Poirot *et al.*, 2004 ▶; Notredame *et al.*, 2000 ▶), which incorporates structural information into multiple sequence alignments. The sequence order was kept as input. Secondary-structure assignment was performed with *DSSP* (Kabsch & Sander, 1983 ▶) using chains *A*, *B* and *C* (VP1, VP2 and VP3, respectively) from the atomic model of TrV as input and it was overlaid on top of the alignment using the *ESPript* software (Gouet *et al.*, 1999 ▶). For VP4, these calculations were based on the theoretical model.

### 2.4. Close-range salt-bridge analysis   

For this study, the reference protomer and all of its symmetry-related equivalents within a distance of 50 Å were analyzed. Symmetry mates were generated with *O* (Jones *et al.*, 1991 ▶) and edited with *MOLEMAN*2 (Kleywegt & Jones, 1994 ▶). Salt bridges were then calculated using the *WHAT IF* software (Vriend, 1990 ▶). All interactions with a distance greater than 4 Å were discarded (Kumar & Nussinov, 2002 ▶).

### 2.5. Insect inoculation and infectivity test of crystallized TrV virions   

All triatomines were immobilized using crossed needles over an expanded polystyrene surface configured to effectively restrict the movement of the insects without injuring them. Prior to inoculation of the liquid, the target surface was rinsed with ethanol in order to prevent bacterial infection. Each perforation was made in a side-most part of the insect to avoid damaging internal organs. All inoculations were performed using sterile 20 µl Hamilton 701-NA syringes. After injecting the liquid, the perforation was immediately sealed using molten paraffin wax. All insects were successfully checked for the absence of any mechanical damage caused by the needle.

Type II crystals were harvested from the growing drop with a CryoLoop, rinsed once for a few seconds in a 5 µl drop of precipitant solution (consisting of 6% PEG 8000, 5% 2-­propanol, 500 m*M* NaCl, 100 m*M* sodium citrate pH 5.6) and then dissolved in a volume of NMT buffer to reach a final virion concentration of 2.7 mg ml^−1^ (the protein content was determined using a BCA assay kit; Pierce). This initial virus solution was diluted 1:3 in NMT buffer and 3 µl aliquots were inoculated into the haemocoel of each of the tested insects (two groups of ten adult *T. infestans* insects each). A double-control test (composed of five adult *T. infestans* insects each) was carried out using 3 µl of NMT buffer on one side and 3 µl of sterile saline solution on the other side as inoculums. After inoculation, all tested insects were examined every 18 h for the first 2 d and daily thereafter to detect symptoms of infection and/or death.

## 3. Results and discussion   

### 3.1. The TrV capsid   

**Figure 1:**
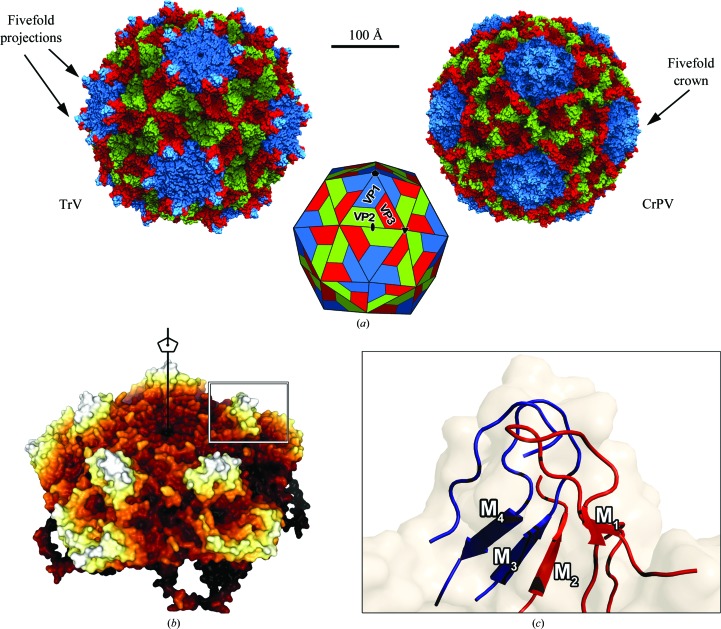
(*a*) Molecular surfaces of *Triatoma virus* (TrV) and *Cricket paralysis virus* (CrPV; PDB entry 1b35). The individual proteins are coloured according to the following code: VP1, blue; VP2, green; VP3, red. The structures are on the same scale. TrV displays characteristic surface projections formed by VP1 and VP3 around the fivefold axes, while there is a depression at the twofold axes of TrV. (*b*) Surface of a TrV penton. A projection has been highlighted in a rectangle. Darker colours correspond to regions closer to the centre of the particle. (*c*) Close-up view of a projection. Loops from VP1 (blue) and VP3 (red) form the projections, with underlying β-sheets. These regions have been identified as M_1_, M_2_, M_3_ and M_4_. The figures in (*a*) were produced with *DINO* (http://www.dino.org) using as input pre-computed surfaces previously calculated with *MSMS* (Sanner *et al.*, 1996 ▶)

**Figure 2:**
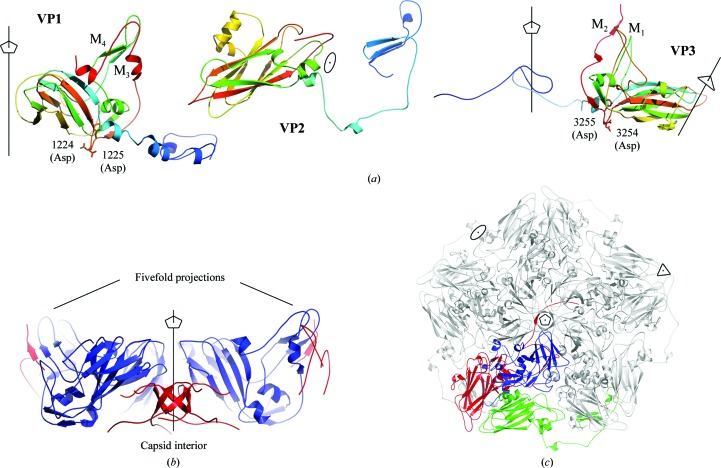
TrV proteins VP1, VP2 and VP3 and relation to the symmetry axes of the capsid. (*a*) Ribbon diagrams colour-ramped from the N-terminus to the C-­terminus: blue to red through yellow. All three proteins adopt the standard jelly-roll topology and have their N-termini lying in the particle interior. The symmetry axes of the particle are represented as thin black lines, with a pentagon (fivefold), ellipse (twofold) and triangle (threefold) denoting the respective icoshahedral axes of the particle. The proposed proteolytic residues (1223–1225 and 3254–3256; see text) are also depicted. (*b*, *c*) Virion assembly. The TrV capsid proteins and their interactions about the fivefold axes. (*b*) Simplified lateral view. VP1 (blue) and only part of VP3 (red) is shown for clarity. The N-tails of VP3 intertwine around the fivefold axis to form a β-cylinder, while two loops and a β-sheet join VP1 in the formation of the projections. (*c*) Top view of a penton (a pentamer of protomers), showing the presence of a channel with a minimum aperture of 6 Å and the extended conformation of the VP3 N-termini surrounding the fivefold axes (VP1, blue; VP2, red, VP3, green).

**Figure 3:**
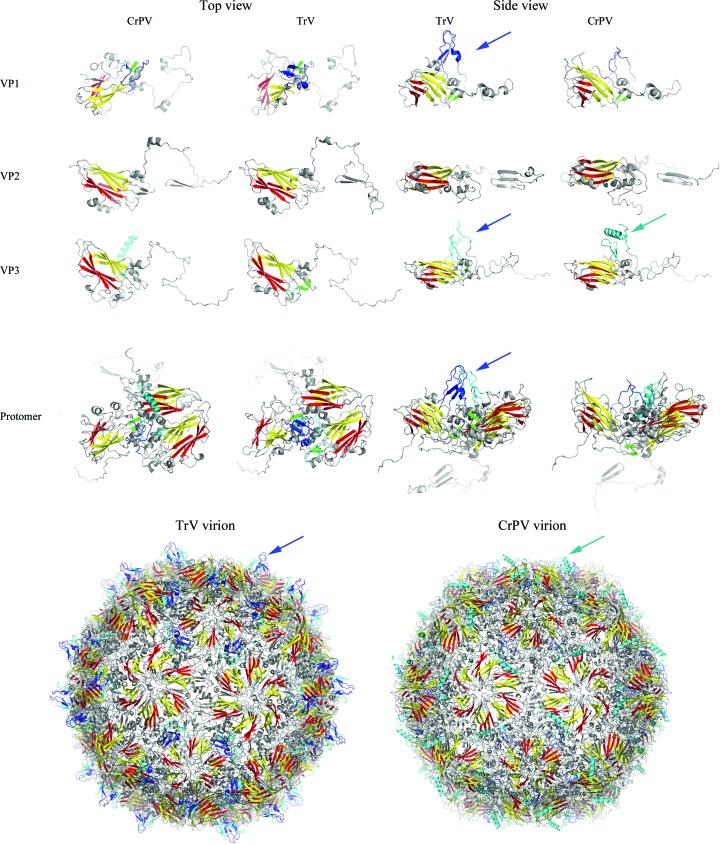
Side-by-side comparison of the TrV and CrPV capsid proteins. CrPV VP1, VP2 and VP3 were superposed on the jelly rolls of their TrV counterparts and are shown side by side in two orthogonal views. Note the striking similarity of the path of the N-tails, despite an amino-acid sequence conservation of only about 20%. The *CHEF* and *BIDG* β-sheets of the jelly rolls are coloured red and yellow, respectively (note the way that the two β-sheets alternate about the fivefold and quasi-sixfold axes of the particles in the bottom row). VP1 segments involved in the projections by the fivefold axes are shown in blue and those from VP3 are shown in cyan. These features are coloured identically in the structural alignment (Fig. 5 ▶). Note that the exposed surface features are quite different in the two viruses despite the remarkable structural conservation of the capsid framework.

The virion was crystallized in two crystal forms, rhombohedral and orthorhombic, which diffracted to 3.2 and 2.5 Å resolution, respectively, as reported previously (Rozas-Dennis *et al.*, 2004 ▶). The structure was determined by molecular replacement (see §[Sec sec2]2) and the initial interpretable electron-density map was improved by real-space averaging using a combination of both crystal forms. The final model was traced and refined to 2.5 Å resolution against the diffraction data from the orthorhombic crystals (Table 1 ▶). The final refined model accounts for the polypeptide chain of three of the four expected proteins in the capsid, without the small protein VP4 (5.5 kDa). Also, in the final model VP1 (271 amino acids) lacks seven C-terminal residues, VP2 (255 amino acids) lacks eight N-terminal residues, and VP3 (285 amino acids) lacks nine C-terminal residues which are disordered in the capsid. In enteroviruses and in CrPV, the residues are numbered from 1001, 2001, 3001 and 4001 for VP1, VP2, VP3 and VP4, respectively, and we also follow this convention in this manuscript. The electron density was interpreted according to the published amino-acid sequence translated from the deposited genomic nucleotide sequence (GenBank accession No. AF178440; Czibener *et al.*, 2000 ▶), with a minor discrepancy in VP3 residue 3054, which corresponds to valine in the sequence but displays an electron density that is more compatible with methionine. Taking into account that this residue is conserved as methionine in all of the other dicistroviruses that we have analyzed, this difference probably arises from an error in the original sequencing (Czibener *et al.*, 2000 ▶). Overall, the TrV capsid displays icosahedral symmetry with pseudo-triangulation number *P* = 3 (Fig. 1 ▶), as expected. It is built from 60 protomers made up of proteins VP1, VP2 and VP3. VP1 is located around the fivefold axes, and VP2 and VP3 alternate around the threefold icosahedral axes (Figs. 2 ▶ and 3 ▶). The three capsid proteins display the standard core domain folded as an eight-stranded antiparallel jelly-roll β-barrel. This β-­barrel, which is composed of opposing β-sheets *BIDG* and *CHEF* (coloured red and yellow, respectively, in Figs. 3, 5 and 6), is such that the β-sheets alternate around the fivefold axes (VP1) and around the quasi-sixfold axes (VP2 and VP3). This feature, highlighted in Fig. 3 ▶, is observed in all quasi-equivalent RNA virus particles that are based on a jelly-roll motif. In addition, each β-barrel has N-terminal and C-terminal arms that intertwine to make internal and external surface features, as well as intra-protomer and inter-protomer inter­actions.

As previously described in the comparison between the capsids of CrPV and the enteroviruses (Tate *et al.*, 1999 ▶), there is neither a canyon nor a pocket in the TrV particle (Supplementary Fig. S1^1^). In contrast to CrPV, TrV displays relatively pronounced projections at the external surface of the particle surrounding the fivefold axes (Figs. 1 ▶ and 2 ▶ *a*). The projections are composed of loops and β-sheets from VP1 and VP3 (highlighted in blue and cyan, respectively, in Figs. 3, 5 and 6) in regions in which there are specific insertions in TrV (M_1_–M_4_ in Figs. 1, 2 and 5). These surface features, which were also apparent in our previous low-resolution cryo-EM reconstructions (Agirre *et al.*, 2013 ▶; Estrozi *et al.*, 2008 ▶), result in a slightly larger maximum diameter (33.51 *versus* 32.92 nm) in the TrV particle despite it having about 4% fewer residues. At the fivefold axis there is a channel of roughly 10 Å in diameter with residues 1128 (Gln), 1166 (Val) and 1167 (Thr) lining its inner wall. The channel is blocked by a density feature that can be interpreted as a metal ion coordinated by the side chain of Gln3014 and its fivefold counterparts. An uninterpreted peanut-shaped density feature is also found below the metal. These features were all observed in a map calculated from data in the resolution range 8–2.5 Å and inspected at 2σ in order to discard low-resolution artifacts accumulated on the symmetry axis.

Both VP2 and VP3 have extended N-­terminal tails (called N-tails below), which are very similar to those observed in the corresponding CrPV proteins (Fig. 3 ▶). In particular, the VP2 N-tail exchanges with its twofold symmetry-related neighbours, resulting in strand swapping (Bennett *et al.*, 1995 ▶), which was one of the characteristic features of dicistroviruses as concluded from the comparison of the CrPV particle with those of the picornaviruses (Tate *et al.*, 1999 ▶). This organization allows VP2 to interact with distant VP1 and VP3 copies from other protomers (Table 2 ▶) and further stabilizes the VP2 dimer. The strand-swapping mechanism has also been observed in several plant viruses (Qu *et al.*, 2000 ▶) and oligomeric proteins (Schlunegger *et al.*, 1997 ▶; Bennett *et al.*, 1994 ▶), but not in the picornaviruses, as mentioned above. The conservation of the amino acids involved in the strand swap across the *Dicistroviridae* family predicts that this feature is present across the whole virus family, as suggested previously (Liljas *et al.*, 2002 ▶).

The VP3 N-tails intertwine around the fivefold axes of the particle, just like the CrPV counterparts, creating a β-cylinder of 6 Å in diameter right beneath the pore present between five copies of VP1 (Fig. 2 ▶ *b*). Two salt bridges link each VP3 N-tail with a VP2 copy from another protomer. It is notable that the presence of multiple interprotomer salt bridges contrasts with the single salt bridge occurring within a protomer (Table 2 ▶); this may have implications in virus assembly and uncoating.

### 3.2. VP4 is disordered within the TrV capsid   

In picornaviruses, the smallest structural protein VP4 (of about 5–7 kDa) plays an important role during cell entry (Hewat *et al.*, 2002 ▶; Schneemann *et al.*, 1992 ▶). Although the N-­terminal end of VP4 is disordered in several picornaviruses (Hadfield *et al.*, 1997 ▶; Kim *et al.*, 1989 ▶; Zhao *et al.*, 1996 ▶), it is found to be well structured at the capsid interior in most of them (Hogle *et al.*, 1985 ▶; Verdaguer *et al.*, 2000 ▶). In particular, in the human rhinovirus VP4 adopts a β-cylinder conformation around the fivefold axis (Hadfield *et al.*, 1997 ▶). In the CrPV crystal structure VP4 displays a compact pentameric α-annulus centred on the fivefold axis (Tate *et al.*, 1999 ▶). A number of conformational differences between VP4 in CrPV with respect to its picornavirus counterpart suggest that VP4 in the insect viruses may not play the same role as it does in picornaviruses (Rossmann & Tao, 1999 ▶; Tate *et al.*, 1999 ▶). VP4 is known to be present in infectious TrV particles (Agirre *et al.*, 2011 ▶), but its location within the capsid has remained elusive not only by X-­ray crystallography but also by two different cryo-EM three-dimensional reconstruction methodologies (Agirre *et al.*, 2013 ▶; Estrozi *et al.*, 2008 ▶). These data suggest that VP4 is present in TrV but is not icosahedrally ordered.

To confirm that the virus particles in the crystals are still infectious, we performed an infectivity test with a solution of redissolved TrV crystals. All ten insects inoculated with the solution from dissolved crystals died in 18 h, while only two insects out of the ten in the uninfected control set died after 15 d (data not shown), confirming that the crystallization process does not affect the infectivity of the virus particles.

**Figure 4:**
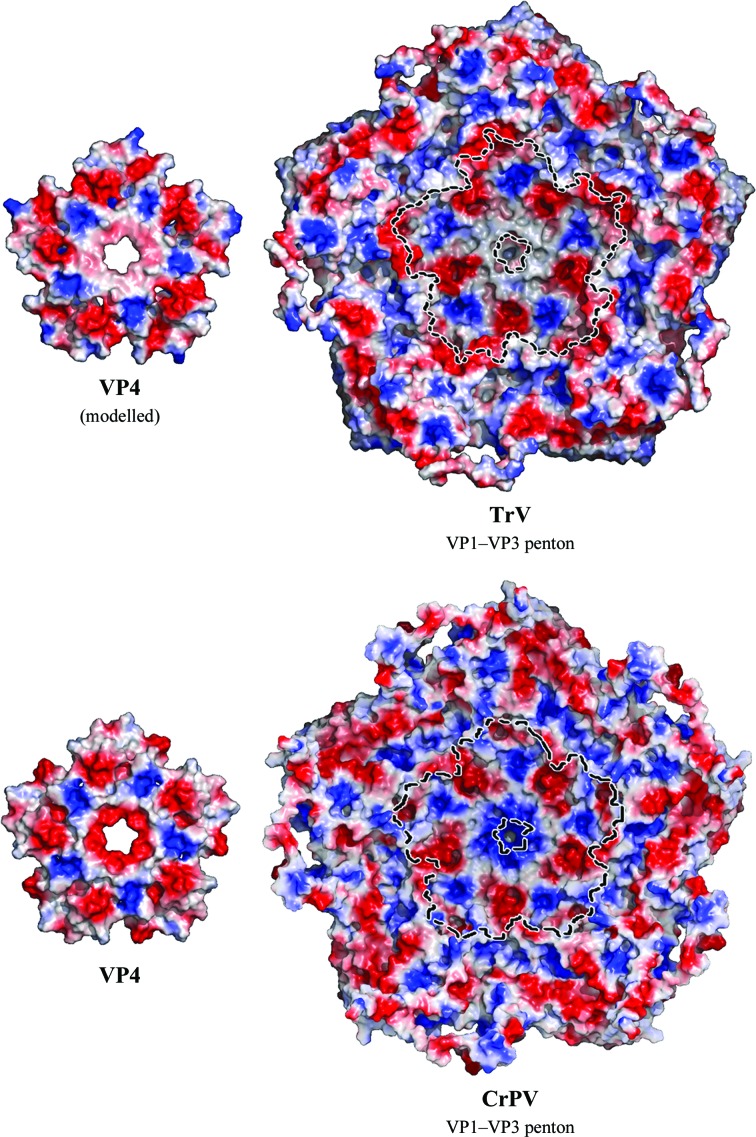
Putative interaction site of VP4 in TrV. Open-book view of the surface electrostatic potential of VP4 and the rest of a penton. The charge distribution appears to be complementary in both cases, except at the centre, on the fivefold axis. The residues that confer opposite charges to this region in CrPV are Glu4009 and Lys3008, which form a salt bridge that cannot occur in TrV. This may be one of the reasons why VP4 is not icosahedrally ordered in the TrV capsid.

**Figure 5:**
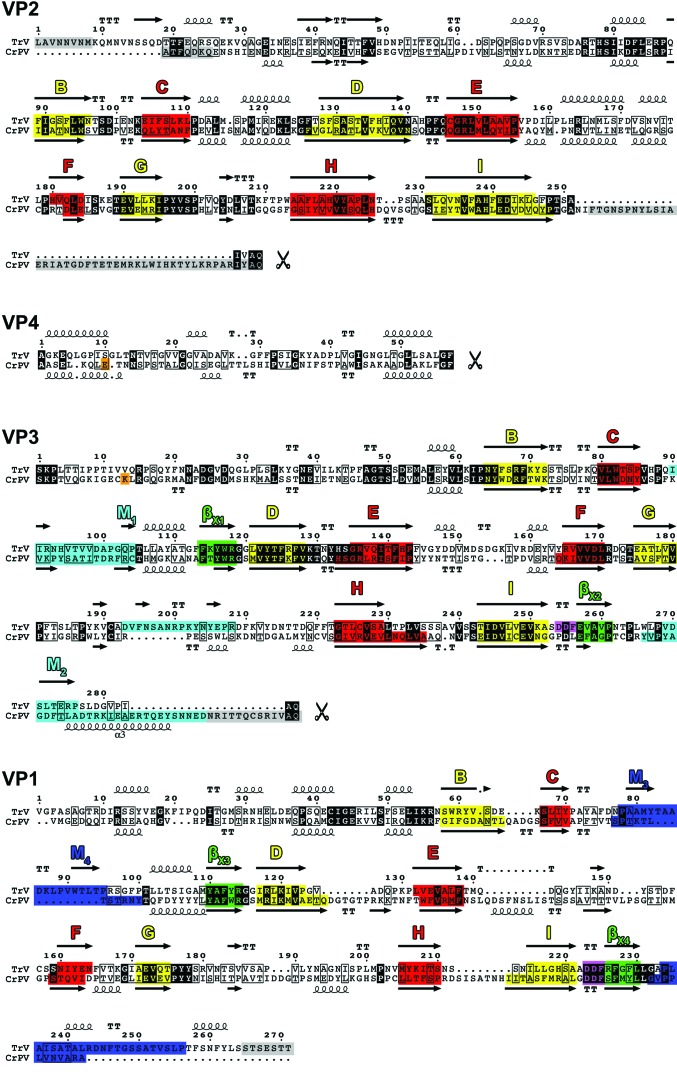
Structural alignment of TrV and CrPV. The colour scheme is as in Fig. 3 ▶. β-Strands are marked with arrows above the sequences and labelled. Spirals denote 3_10_-helices and α-helices. Regions involved in the formation of the projections by the fivefold axes are highlighted in dark blue (including strands M_3_ and M_4_ in VP1) and cyan (including M_1_ and M_2_) in VP3. β-Strands in the *CHEF* and *BIDG* β-sheets of the jelly roll are shown in red and yellow, respectively. The DDF motifs are indicated in purple. The β-strands that link to the DDF motifs are coloured green (β_x3_β_x4_ in VP1 and β_x1_β_x2_ in VP3). In CrPV, the residues involved in an inter-protomer salt bridge between VP4 and VP3 are highlighted in orange. Conserved residues are highlighted in white font on a black background and similar residues in both sequences are framed. A grey background denotes residues disordered in the structure. This figure was prepared with *ESPript* (Gouet *et al.*, 1999 ▶)

Homology modelling of TrV VP4 using the CrPV VP4 pentamer formed about the icosahedral fivefold axis as a template (see §[Sec sec2]2) resulted, as expected, in a structure displaying the main features of the α-annulus adopted by the template (Fig. 4 ▶). Analysis of this model, after superposition of the TrV and CrPV pentamers, revealed that the TrV VP4 ring displays an electrostatic charge distribution that is complementary to that of the surrounding capsid when placed at the location indicated by the CrPV structure. This outcome allows us to hypothesize that, despite the low sequence identity (19.2% calculated from the alignment supplied as input to *MODELLER*), TrV VP4 might indeed adopt a similar structure to that of CrPV VP4. A detailed analysis of the CrPV atomic contacts along with their calculated binding and solvation energies was conducted using precalculated data available from ViperDB (Carrillo-Tripp *et al.*, 2009 ▶) and revealed that amino acids 4009 (Glu) and 3008 (Lys) make five interprotomer salt bridges between the VP4 α-annulus and the capsid interior. While it only accounts for 10% of the total calculated solvation energy for the interface between VP4 and the rest of the capsid, this salt bridge remains the only specific interaction, with the other interactions being of hydrophobic nature. However, the residues that participate in the salt bridge are not conserved in TrV (residues annotated in orange in Fig. 5 ▶) and the absence of this interaction could result in disorder or even the dissociation of VP4 from the internal TrV capsid surface. We note also that the opposite face of the modeled α-annulus, which would be facing the genomic RNA, has a uniform basic surface electrostatic potential (data not shown). TrV VP4 might therefore bind more strongly to the negatively charged RNA and these interactions could play a role in displacing it from its initial location in an immature capsid, where it is derived by autocleavage of a VP0 precursor. Amino-acid sequence comparisons (Supplementary Fig. S3) suggest that a subgroup of the dicistroviruses, including *Plautia stali intestine virus* (PSIV), *Himetobi P virus* (HiPV) and *Black queen cell virus* (BQCV), might also have a disordered VP4 in their capsid, since they also lack the residues that make a salt bridge in CrPV. These viruses have been proposed along with TrV to be part of the genus tentatively named *Triatovirus* (Agirre *et al.*, 2011 ▶).

### 3.3. A second DDF proteolytic motif in the TrV capsid   

As introduced above, in the case of CrPV the amino-acid triad DDF (amino acids 1241–1243), which is located after strand *I* of the VP1 jelly roll, has been proposed to be responsible for the cleavage of VP0 into VP4 and VP3 upon capsid assembly (Tate *et al.*, 1999 ▶). The side chain of the first aspartic acid in the motif points towards the gap between VP4 and VP3 from an adjacent fivefold-related protomer, suggesting that this cleavage step can only take place after at least a penton has been formed during assembly of the virion.

**Figure 6:**
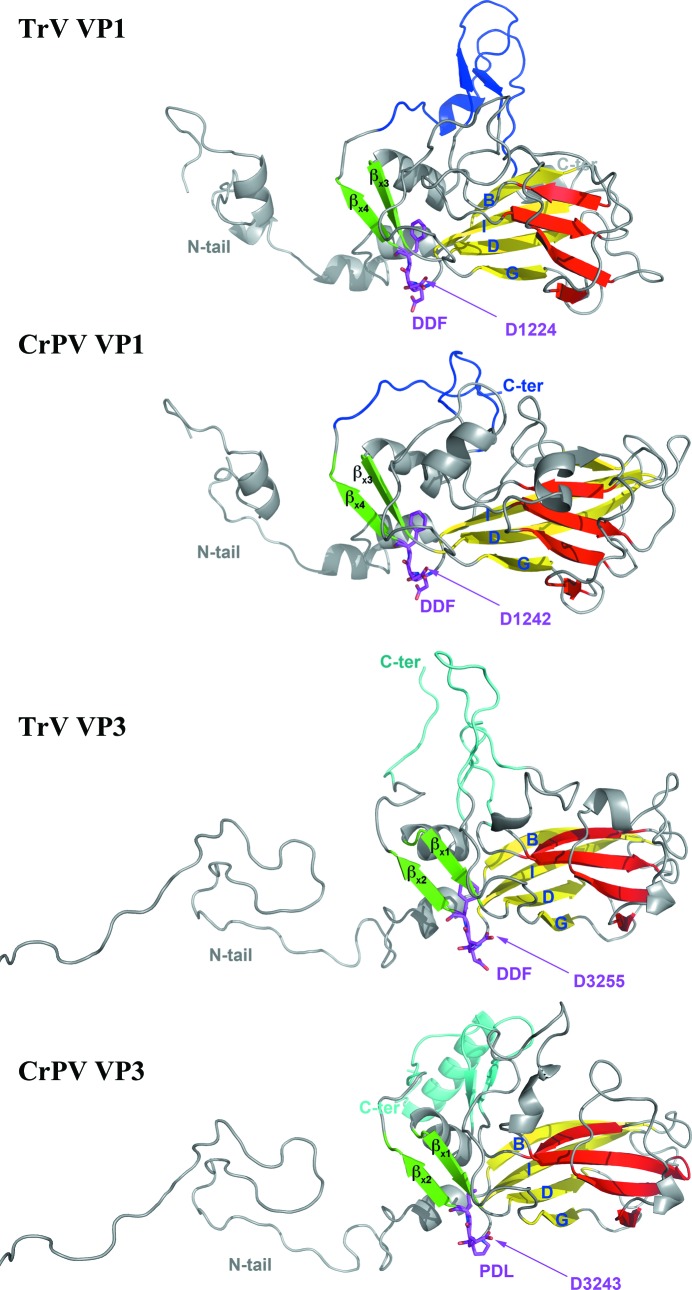
The DDF catalytic triad. The colour code is the same as that in Figs. 3 and 5. This figure highlights the structural conservation of the DDF triad in VP1 and in VP3, in both cases exactly in the same location with respect to the jelly roll and the small β-sheets shown in green. The first aspartic acid, which points into the interior of the particle, is not conserved in CrPV VP3, in which it is proline. The conservation of the second aspartic acid (indicated by the arrows) is likely to be a consequence of structural constraints (see text). The conservation of the DDF motif in the same location in VP1 and in VP3 in several dicistroviruses is intriguing, given that the two proteins have only 12% sequence identity and have diverged substantially otherwise.

In TrV, the DDF triad (Fig. 2 ▶; amino acids 1223–1225) occupies exactly the same location, with main-chain and side-chain con­formations exactly matching those observed in the CrPV capsid. The VP3 N-terminus, which is derived from the cleavage of VP0 into VP4 and VP3, also superimposes on its counterpart in CrPV. Thus, despite the absence of ordered density for VP4, the organization of the precursor VP0 within the capsid is very likely to be the same as in CrPV. The observation that naturally occurring empty TrV capsids contain unprocessed VP0 (Agirre *et al.*, 2011 ▶) suggests that capsid autocleavage requires RNA encapsidation, in addition to having the catalytic residue in the vicinity of the VP0 scissile bond. The VP1 DDF motif is highly conserved, being present in nearly all *Dicistroviridae* members, highlighting its essential role within this virus family (Liljas *et al.*, 2002 ▶). We note that in both CrPV and TrV VP1 the DDF motif is in a segment connecting the end of strand *I* of the jelly roll to a β-strand immediately downstream (termed β_x4_ in Fig. 5 ▶). This second strand makes an antiparallel interaction with a second β-­strand (β_x3_) that precedes strand *D* on the same *BIDG* sheet of the jelly roll. This results in the DDF motif being located in a corner between the *BIDG* and the β_x3_β_x4_ sheets in a bend of about 90° since these two β-sheets are nearly orthogonal to each other, as illustrated in Fig. 6 ▶.

**Figure 7:**
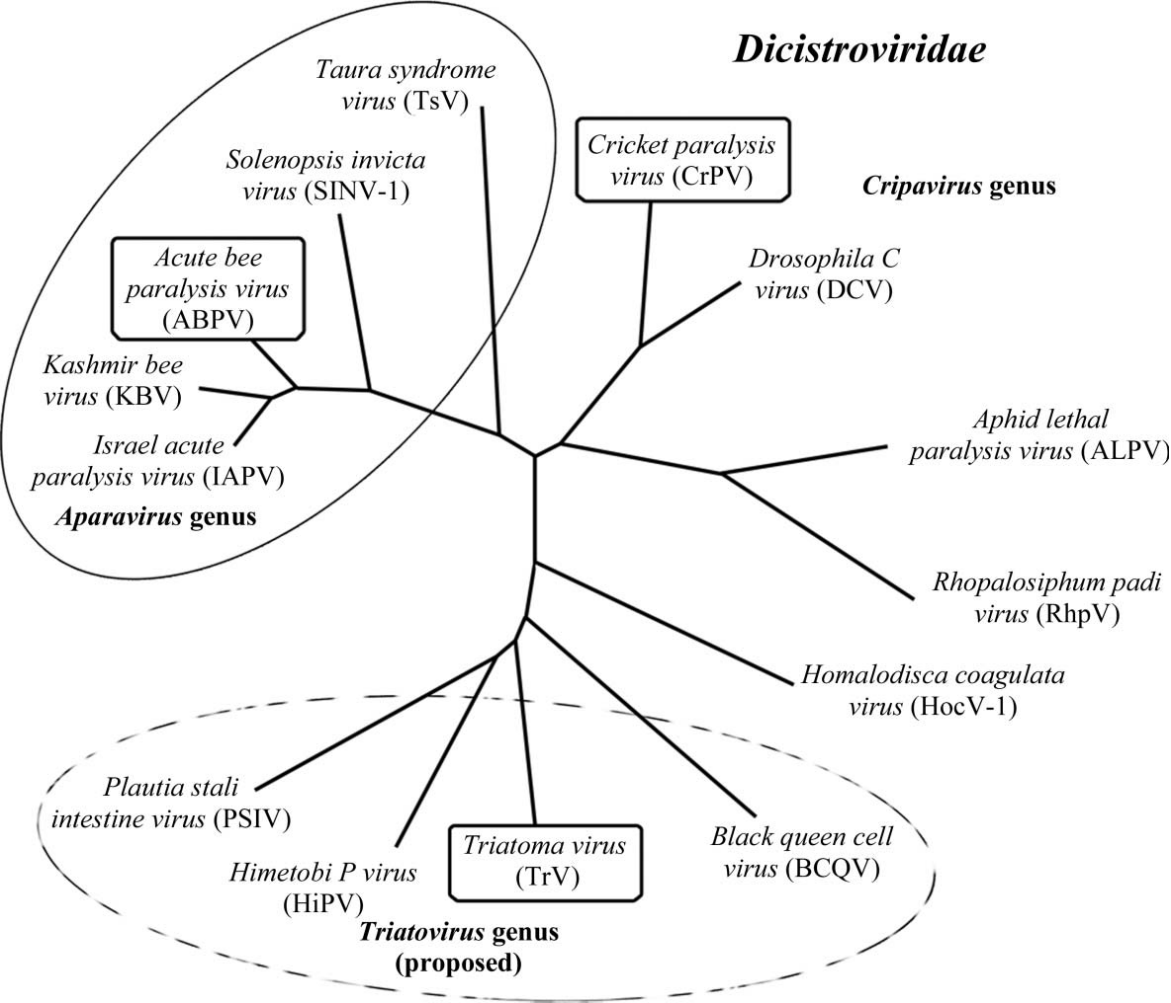
Phylogenetic classification of the available *Dicistroviridae* genome sequences constructed using the *MEGA*5 software (Tamura *et al.*, 2011 ▶). The type species of each genus is boxed. Two genera have been accepted so far: *Cripavirus* and *Aparavirus* (continuous ellipse). The proposed *Triatovirus* genus (Agirre *et al.*, 2011 ▶) would initially cover PSIV, HiPV, BQCV and TrV (dashed ellipse), with TrV as the type species.

Intriguingly, a second DDF motif (3254–3256) is present in VP3 at the same location in the structure at the corner between the *BIDG* sheet and the β_x1_β_x2_ sheet, which is equivalent to the β_x3_β_x4_ sheet in VP1 (Fig. 6 ▶; see also Fig. 5 ▶, in which these β-­sheets are highlighted in green). This motif is also conserved (Supplementary Fig. 3 ▶) in *Black queen cell virus* (BQCV), *Himetobi P virus* (HiPV) and *Plautia stali intestine virus* (PSIV), but is not found in CrPV or in *Drosophila C virus* (DCV), which is the closest neighbour to CrPV in the current *Dicistroviridae* phylogenetic tree (Fig. 7 ▶). Although the motif is not conserved in CrPV, the second aspartic acid in the sequence, Asp3243, is conserved and has the same orientation in the structure, with the side chain accepting a hydrogen bond from the main-chain amide of a conserved glycine residue between strands β_x1_ and *D* in VP3 and between strands β_x3_ and *D* in VP1. In the case of TrV, the side chain of Asp3255 (the second aspartic acid in the motif) also accepts a hydrogen bond from the main-chain amide of residue 3040 in the N-tail of the same VP3 molecule. This suggests that the second aspartic acid of the DDF motif is conserved because it plays a structural role and is not likely to have a catalytic role, leaving the first aspartic acid as the likely candidate. In contrast to the VP1 DDF motifs, in which the putative catalytic aspartic acid points to the N-terminus of VP3, in TrV VP3 this residue points towards the polypeptide segment spanning amino acids 1032–1035 in the VP1 N-tail. This segment is not cleaved, although it is plausible that an RNA rearrangement prior to exit may activate cleavage at this location. If this were the case, it would mean that TrV and CrPV have a different mechanism for RNA release, since the proline residue present in CrPV VP3 at the position of the catalytic aspartic acid would not be functional. The striking similarity between VP1 and VP3 in the region of the DDF motif may be a remnant of the particular evolutionary path of *P* = 3 viruses, which is postulated to have taken place *via* successive gene duplications from an ancestral precursor displaying *T* = 3 symmetry (Liljas *et al.*, 2002 ▶). However, it is intriguing that the motif is conserved between VP1 and VP3, which share only about 12% sequence identity. In this respect, it is noteworthy that when comparing TrV VP1 with the protein database with the *DALI* server (Holm & Rosenström, 2010 ▶), the highest score is for CrPV VP1 as expected, but picornavirus VP3 is close behind, suggesting a relatively close relation between the two proteins. In contrast, when looking at VP2, the closest match after CrPV is the jelly roll from the capsid protein of *Norwalk virus*, which is not a picornavirus and exhibits *T* = 3 symmetry. It is of course impossible to define an evolutionary path from these data, but it serves to highlight how intriguing the conservation of the DDF triad in VP3 and VP1 within the same structural motif is.

## 4. Conclusions   

In this work, we analyzed the structure of the TrV capsid and compared it with that of CrPV. These are the only members of the *Dicistroviridae* family for which the capsid structure is known at atomic resolution. The salient capsid features that differentiate dicistroviruses from enteroviruses in the *Picornaviridae* family are the absence of both the canyon and the pocket factor, and the swapping of the VP2 N-tail. The absence of the canyon on the TrV and CrPV capsid surfaces strongly suggests a different approach to receptor binding to that of the majority of picornaviruses. TrV and CrPV also lack the characteristic enterovirus pocket factor found in the interior of the VP1 β-­barrel; this implies that capsid stabilization in both of the insect viruses differs from that in most picornaviruses. The domain swapping in both TrV and CrPV, through the twofold axis of the icosahedral capsid, suggests that the assembly mechanism in dicistroviruses may be different to that in picornaviruses, which occurs through pentameric intermediates (Rossmann & Tao, 1999 ▶).

The two main differences that we observe between the TrV and CrPV capsids are the projections at the TrV surface, which are absent in CrPV. These projections are made from an inserted sequence element, and the presence of the same insertion in other members of the proposed genus *Triatovirus* (Agirre *et al.*, 2011 ▶) suggest that they are also likely to have the same structural features. The structure suggests that the exposed residues in these projections are likely to play a role in the interactions with the host; for instance, an entry receptor. Knowledge of this feature therefore now opens the way to site-directed mutagenesis in order to understand the functional importance of the residues that are exposed at the projections.

The second difference is the presence of a DDF motif in the homologous location in VP3, where it is also exposed to the capsid interior, whereas in CrPV this motif is only found in VP1, where it is believed to account for cleavage of the VP0 precursor. This difference may account for additional proteolysis in VP1 during RNA release, as discussed above, but further studies are necessary in order to test this hypothesis.

Finally, the most striking difference is the absence of ordered VP4. The fact that this protein is not part of the ordered capsid in fully infectious TrV virions is in sharp contrast to what occurs with the same protein in CrPV, where it is found to be well structured around the fivefold axis. Such differences preclude assignment of a general function to VP4 in the *Dicistroviridae* family, and this also remains to be further elucidated.

To conclude, the new structural data presented here now open the way for functional studies to understand the important steps of the TrV cycle, such as its assembly during exit and uncoating during entry. If this virus is to be used as a biological control against Chagas disease, the more knowledge that we can gather about it the better prepared we will be to overcome the inherent difficulties that are related to this type of approach.

The atomic coordinates and structure factors for TrV have been deposited in the Protein Data Bank (http://www.pdb.org) as entry 3nap. The theoretical model for VP4 has been deposited in the Protein Model DataBase (http://mi.caspur.it/PMDB) with code PM0077751.

## Supplementary Material

PDB reference: TrV, 3nap

Supporting information file. DOI: 10.1107/S0907444913004617/mn5025sup1.pdf

## Figures and Tables

**Table 1:** Data-collection and refinement statistics Values in parentheses are for the highest resolution shell.

Diffraction data	
Diffraction source	ESRF beamline ID23-1
Detector	MAR Mosaic 225mm CCD
Temperature (K)	100
Space group	*P*2_1_2_1_2
Unit-cell parameters (, )	*a* = 343.7, *b* = 360.5, *c* = 341.3, = = = 90
Resolution range ()	37.92.5 (2.632.50)
Measured reflections	5629383 (309870)
Completeness %)	92.4 (74.0)
*R*_merge_ (%)	12.9 (32.8)
*I*/(*I*)	4.4 (2.1)
Wavelength ()	0.93
Multiplicity	4.2 (2.0)
Overall *B* factor from Wilson plot (^2^)	19.8
Refinement statistics	
Content of the asymmetric unit	1/2 of virus particle
Resolution range ()	30.02.5
No. of non-H atoms	
Protein	6196
Ligand	0
Water	553
Total	6749
R.m.s. deviations	
Bonds ()	0.013
Angles ()	1.9
Average *B* factors (A^2^)	
Protein	22.0
Water	32.4
Ramachandran plot	
Favoured regions (%)	96.7
Additionally allowed (%)	2.8
Outliers (%)	0.4
*R*_cryst_/*R*_free_ † (%)	17.12/17.32

† *R*_cryst_ = 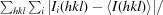 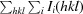. *R*_free_ is the same as *R*_cryst_ but was calculated using 1% of reflections not used in refinement.

**Table 2:** Summary of the salt bridges present in the TrV structure Intramolecular bridges occur within the same protein in each protomer. Intraprotomer bridges occur between two amino acids from different proteins within a protomer. Interprotomer bridges occur between two amino acids from proteins in different protomers. The displayed distances (4) were measured between the closest pairs of interacting atoms.

	Salt bridges		
	Intramolecular	Intraprotomer	Interprotomer
VP1	Glu1033Arg1030 (3.0)	Asp1010Lys3251 (3.1)	Glu1033Arg2070 (3.7)
	Asp1151Lys1148 (2.9)		Asp1035Arg2070 (3.9)
	Asp1155Lys1207 (2.7)		Asp1142Arg3200 (3.6)
	Glu1163Lys1168 (3.2)		Asp1151Arg1243 (2.5)
	Glu1172Lys1120 (3.0)		Glu1163Arg2015 (2.9)
	Glu1172Lys1168 (2.7)		
VP2	Glu2027Lys2028 (3.0)		Glu2022Arg3009 (3.3)
	Asp2074His2078 (2.8)		Glu2027Lys3019 (3.8)
			Glu2033Lys1187 (2.8)
			Glu2121Arg2159 (3.7)
VP3			

## Footnotes

1 Supplementary material has been deposited in the IUCr electronic archive (Reference: MN5025). Services for accessing this material are described at the back of the journal.
