# Supplementary material for: Structure of the Triatoma virus capsid
Source: Acta Crystallogr D Biol Crystallogr. 2013 May 14;69(Pt 6):1026–37. doi: 10.1107/S0907444913004617 (PMC3663122; doi:10.1107/S0907444913004617)
Supplement: Supplementary file 1 [file d-69-01026-sup1.pdf]

# Supplementary Material

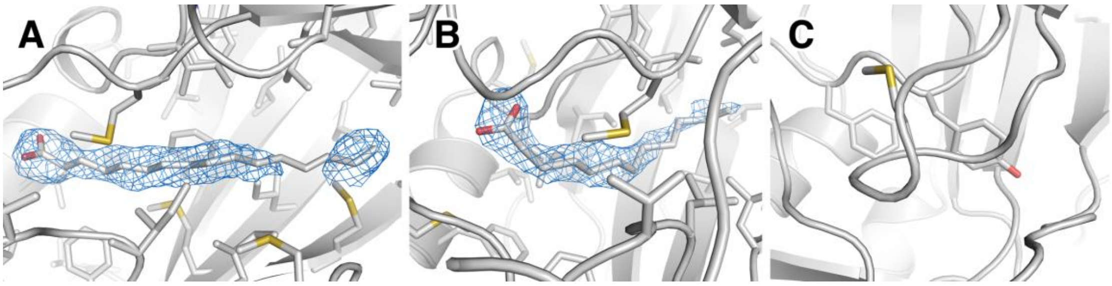


**Supplementary Figure S1. The Pocket Factor in enteroviruses is absent in the TrV capsid.** **A:** the pocket region of Echovirus 1 (PDB 1EV1) (Filman *et al.*, 1998). A 60-fold NCS averaged 2fo-fc map (level: 1.5σ) is displayed containing a palmitate molecule. Only hydrophobic side chains are depicted for clarity. **B:** the same view in Human Rhinovirus 2 (PDB 1FPN) reveals the presence of a lauric acid molecule (Verdaguer *et al.*, 2000). A 15-fold NCS averaged 2fo-fc map (level 1.0σ) is displayed. **C.** The same region in TrV is occupied by a long loop and by both large and hydrophobic side chains. The three structures were superimposed by the main chain atoms in order to produce the closest views.

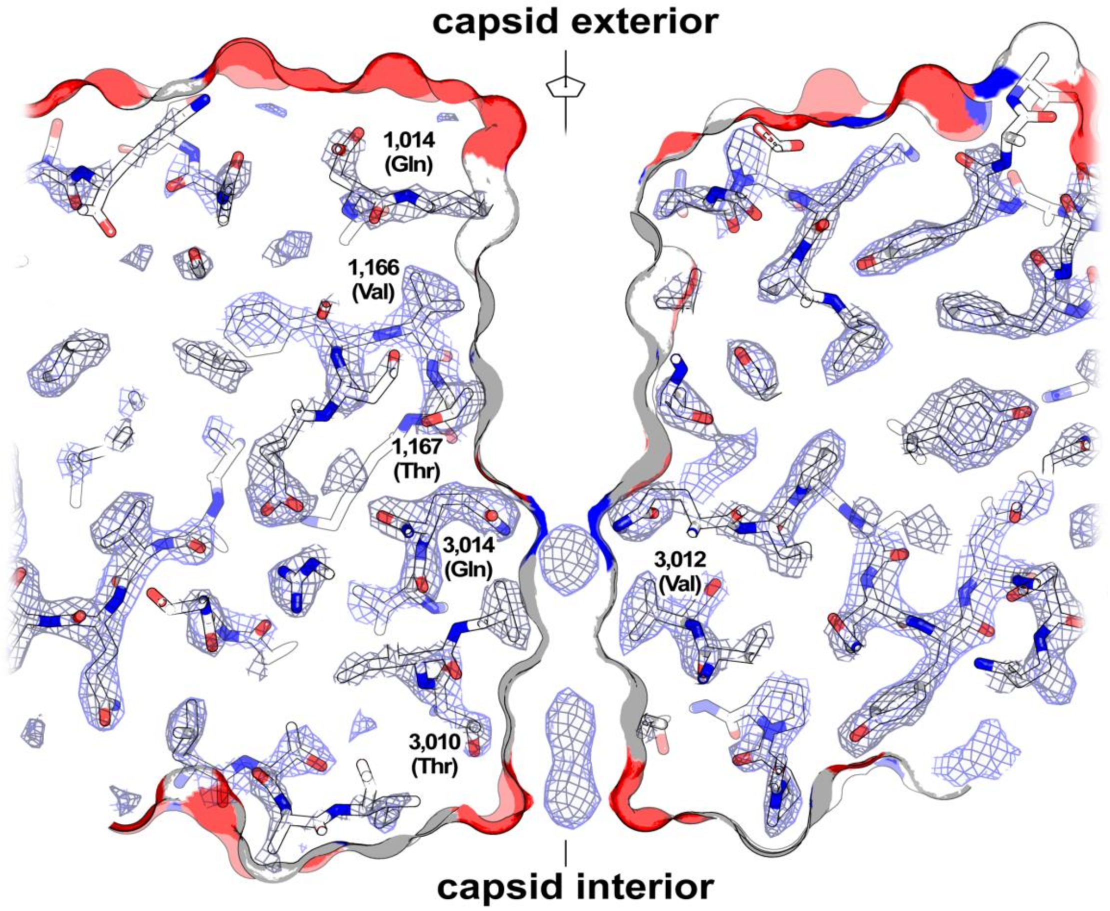


**Supplementary Figure S2. Slab containing the 5-fold axis of the TrV capsid.** A 30-fold NCS averaged 2fo-fc map (level: 2.0σ) shows the electron densities populating the channel formed between VP1 and VP3. The surface represented corresponds to the solvent accesible area, and has been colored accordingly to the charges from neighboring atoms. The outer region of the channel is mainly hydrophobic (colored gray). In the central channel portion, a double ring made of Q1,014, V1,166 and T1,167 traps a round density most likely attributable to a metal ion coordinated by Q3,014. The internal channel portion also contains an uniterpreted peanut shaped density. Note: the electron density along the 5-fold axis was not modeled, so this corresponds to a symmetry averaged omit map.

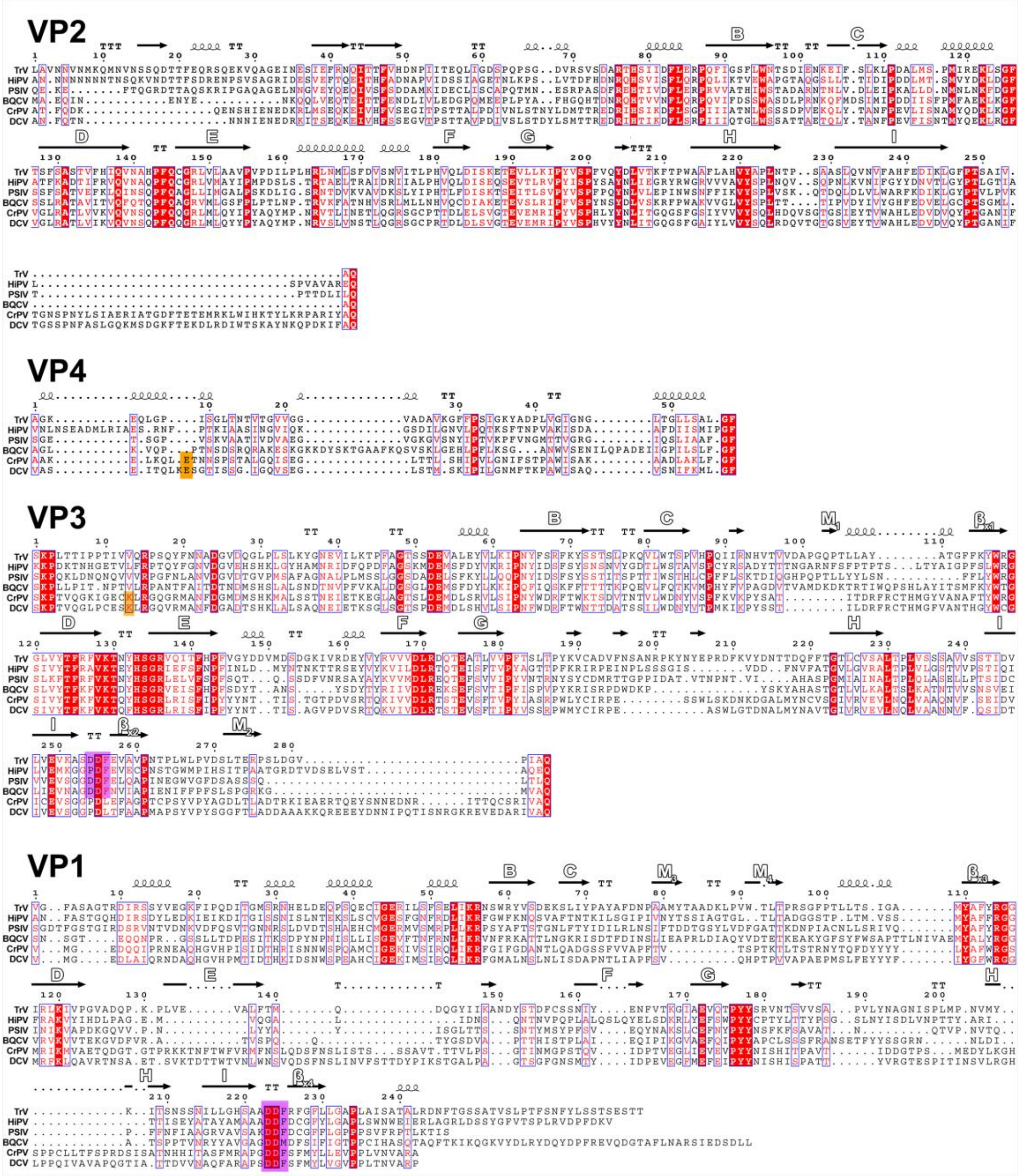

**Supplementary Figure S3.** Multiple sequence alignment of several *Dicistroviridae* members. The DDF motifs are highlighted in purple. Residues involved the formation of a salt bridge between VP4 and VP3 and the corresponding aligned residues from DCV are highlighted in orange. Total sequence conservation has been marked with a red background, while partial conservation is shown with red characters on a white background.
